# Supplementary material for: Mapping the evolution of fertility support policies in China: A content and instrumental analysis
Source: PLoS One. 2025 Oct 9;20(10):e0332137. doi: 10.1371/journal.pone.0332137 (PMC12510515; doi:10.1371/journal.pone.0332137)
Supplement: S1 Appendix — (ZIP) [file pone.0332137.s001.zip › S1 Appendix. 226 original policy documents/189-国家发展改革委办公厅、国家卫生健康委办公厅关于编报普惠托育服务专项行动2020年中央预算内投资项目计划建议的通知(FBM-CLI-4-337787).doc]

国家发展改革委办公厅、国家卫生健康委办公厅关于编报普惠托育服务专项行动2020年中央预算内投资项目计划建议的通知

**发布部门：** [国家发展和改革委员会(含原国家发展计划委员会、原国家计划委员会)](https://www.pkulaw.com/law/chl?Aggs.IssueDepartment=60301) [国家卫生健康委员会](https://www.pkulaw.com/law/chl?Aggs.IssueDepartment=60321) [机构沿革](javascript:void(0))

**发文字号：**发改办社会〔2019〕1090号

**发布日期：**2019.11.26

**实施日期：**2019.11.26

**时效性：** [现行有效](https://www.pkulaw.com/law/chl?Aggs.TimelinessDic=01)

**效力级别：** [部门工作文件](https://www.pkulaw.com/law/chl?Aggs.EffectivenessDic=XE0303)

**法规类别：** [预算管理](https://www.pkulaw.com/law/chl?Aggs.Category=03126)

国家发展改革委办公厅、国家卫生健康委办公厅关于编报普惠托育服务专项行动2020年中央预算内投资项目计划建议的通知
（发改办社会〔2019〕1090号）

各省、自治区、直辖市及计划单列市、新疆生产建设兵团、黑龙江省农垦总局发展改革委、卫生健康委：
　　根据《支持社会力量发展普惠托育服务专项行动实施方案（试行）》（发改社会﹝2019﹞1606号，以下简称《实施方案》），2020年国家发展改革委安排中央预算内投资支持普惠托育服务设施建设。现将编报投资计划建议方案的有关要求通知如下。
　　**一、**总体要求
　　（一）计划建议必须符合《实施方案》及其附件《支持社会力量发展普惠托育服务专项行动项目和资金管理办法（试行）》相关要求，遴选前期工作成熟、具备开工条件的项目，确保投资计划下达后能尽快执行，不符合专项建设规划条件、前期工作不完备的项目一律不纳入安排范围。2020年投资要严格防范由此增加的地方债务风险。
　　（二）要认真落实《中央预算内投资计划编制管理暂行办法》《国家发展改革委关于加强政府投资项目储备编制三年滚动投资计划的通知》《国家发展改革委办公厅关于使用国家重大建设项目库加强项目储备编制三年滚动投资计划有关问题的通知》。所有申报投资计划的项目，必须纳入国家重大建设项目库，确保入库项目信息无误并与纸质文件完全一致。申报投资计划的项目要由“年度投资计划”库推送，并准确填写申请资金年份等信息。
　　（三）上报投资计划建议方案时，应明确项目（法人）单位及项目责任人、日常监管直接责任单位及监管责任人。项目责任人、监管责任人应分别为项目单位、日常监管直接责任单位的有关领导。明确日常监管责任单位及监管责任人，应经有关单位和责任人认可。投资计划将采取切块方式下达，项目（法人）单位先明确为省级发展改革部门业务处室或有关行业主管部门业务处室，日常直接监管责任单位先明确为省级发展改革部门或有关行业管理部门。分解下达投资计划时，应对分解后的具体项目逐一明确项目（法人）单位及项目责任人、日常监管直接责任单位及监管责任人。

　　**二、**工作安排
　　（一）请各地发展改革部门按照《实施方案》要求，提出建议方案，上报国家发展改革委。
　　（二）普惠托育服务专项行动中央预算内投资补助采用切块下达方式，项目名称采用“XX省（自治区、直辖市）2020年支持社会力量发展普惠托育服务项目”，项目建议方案只需上报项目总数、建设总规模、投资总额等内容，不再上报单个具体项目情况等附件材料。
　　（三）请于2019年12月30日前将2020年建议方案正式文件报送国家发展改革委。同时，各地于12月20日前报送建议方案EXCEL电子版。纸质文件所附计划申报表必须从国家重大建设项目库的年度投资计划编报区选择“2020年中央预算内投资计划申报表”进行打印，不得更改，并将建议方案表EXCEL电子版（见附件）以电子邮件形式报送国家发展改革委（社会发展司）。
　　联系方式：
　　苏成钢 010-68502380；
　　贺　婷 010-68502517，heting@ndrc.gov.cn
　　附件：[1.普惠托育服务专项行动2020年中央预算内投资计划建议方案表](https://resources.pkulaw.cn/staticfiles/20191210/11/30/5/9559ef654bc3bd49d381ac00e348d92d.xls)
　　[2.普惠托育服务专项行动2020年中央预算内投资计划绩效目标表](https://resources.pkulaw.cn/staticfiles/20191210/11/31/5/ad5f127f853edf89b07baddc156e298d.doc)

国家发展改革委办公厅
国家卫生健康委办公厅
2019年11月26日

© 北大法宝：（[www.pkulaw.com](https://www.pkulaw.com/)) 专业提供法律信息、法学知识和法律软件领域各类解决方案。北大法宝为您提供丰富的参考资料，正式引用法规条文时请与标准文本核对。
欢迎查看所有[产品和服务](https://www.pkulaw.com/helps/2.html)。法宝快讯：[如何快速找到您需要的检索结果？](https://www.pkulaw.com/helps/70.html)[法宝](https://www.pkulaw.com/helps/69.html)V6有何新特色？

扫描二维码阅读原文

**原文链接：**<https://www.pkulaw.com/chl/c619928fc4ebc77dbdfb.html>
